# Coding with the machines: machine-assisted coding of rare event data

**DOI:** 10.1093/pnasnexus/pgae165

**Published:** 2024-04-30

**Authors:** Henry David Overos, Roman Hlatky, Ojashwi Pathak, Harriet Goers, Jordan Gouws-Dewar, Katy Smith, Keith Padraic Chew, Jóhanna K Birnir, Amy H Liu

**Affiliations:** Government and Politics, University of Maryland at College Park, College Park, MD, USA; Political Science, University of North Texas, Denton, TX, USA; Government and Politics, University of Maryland at College Park, College Park, MD, USA; Government and Politics, University of Maryland at College Park, College Park, MD, USA; Government and Politics, University of Maryland at College Park, College Park, MD, USA; Government, University of Texas at Austin, Austin, TX, USA; School of Politics and Global Studies, Arizona State University, Tempe, AZ, USA; Government and Politics, University of Maryland at College Park, College Park, MD, USA; Government, University of Texas at Austin, Austin, TX, USA

**Keywords:** machine coding, political event data, GPT, BERT, machine learning

## Abstract

While machine coding of data has dramatically advanced in recent years, the literature raises significant concerns about validation of LLM classification showing, for example, that reliability varies greatly by prompt and temperature tuning, across subject areas and tasks—especially in “zero-shot” applications. This paper contributes to the discussion of validation in several different ways. To test the relative performance of supervised and semi-supervised algorithms when coding political data, we compare three models’ performances to each other over multiple iterations for each model and to trained expert coding of data. We also examine changes in performance resulting from prompt engineering and pre-processing of source data. To ameliorate concerns regarding LLM’s pre-training on test data, we assess performance by updating an existing dataset beyond what is publicly available. Overall, we find that only GPT-4 approaches trained expert coders when coding contexts familiar to human coders and codes more consistently across contexts. We conclude by discussing some benefits and drawbacks of machine coding moving forward.

**Significance Statement**

Validation of machine-coded political data remains a challenge. We assess the performance of three models (a semi-supervised, dictionary-based Bayes classifier, BERT, and GPT) relative to human expert coders by coding data previously unavailable for the training of LLMs. GPT outperforms the other models and approximates trained human coders. Furthermore, our exercise highlights new issues in validation including variance in quality of human coding, the task dependent relevance of prompt engineering, and the effect of data type and pre-processing on machine performance metrics.

## Introduction

Machine coding and analysis of textual data have advanced dramatically in the last three decades (1–3). Researchers now use large language models (LLMs)—instead of human coding ((1), p. 28)—to classify complex text whether it is across cases (4) or within a case (5). Indeed, there are scholars who suggest that LLMs—such as GPT—now rival human crowd-sourced coding of data (6) and even expert coding (7). At the same time, there are concerns that reliability varies greatly—not just by prompt and temperature tuning (8)—but also across source data (9) and tasks (10). These concerns are especially pronounced for LLM classifications that rely on “zero-shot”^a^ applications (11). Consequently, researchers call for LLM classifications to be validated (9, 12). The question remains: How do we validate machine-coded data?

This paper makes multiple contributions to the discussion of validation. First, to counteract concerns with contamination—i.e. testing on data that may have been used in the training of LLMs (e.g. GPT)—we assess performance by coding new data that updates an existing dataset beyond what is publicly available. Second, to validate the machine coding, we compare the performance of three models—to each other, iteratively to themselves, and to human-coded, new data. In doing so, we focus on the crucial topic of prompt engineering, with an eye to improving results in zero-shot applications and beyond (3, 13). Third, while our human-coding follows the literature (6, 7, 9) in relying on expert (student) coders, we iteratively trained them to improve their coding output. This, we believe, provides a more realistic—albeit higher—bar for comparing between machine and human coding. Fourth, and related, in contrast to restricting coding to cases where experts have the requisite familiarity (6, 7), we explicitly compare expert-coding performance across familiar and unfamiliar cases to identify differences between human coders and LLMs. Finally, we address the under-explored topic of the effect of task (10) and source data (9) on LLM performance, especially as they relate to prompt engineering.

We first apply a semi-supervised dictionary-based Bayes classifier model to code ethnic group protests across time and space. We use this model to identify rare or difficult-to-categorize topics such as protest—using a well-defined dataset on identity groups. The *All Minorities at Risk* (AMAR) project^b^ documents ethnic minorities worldwide 1945–2006. Today, AMAR (and its predecessor) data are widely used in the study of ethnic politics—from elections to rebellion (14). Using the AMAR framework, we code protest events across ethnic groups in different countries during years not previously included in the data. Next, we apply two LLMs developed for Natural Language Processing (NLP) to code the same data from the same sources. The first model is Bidirectional Encoder Representations from Transformers (BERT). This model uses a transformer mechanism to learn contextual relations between words in both directions of a text simultaneously. The second LLM is the Generative Pre-trained Transformer (GPT). Both BERT and GPT are pre-trained on large volumes of text data, designed to predict the next word in a text, and can be fine-tuned for downstream tasks such as classification.

We begin with a discussion of the data to be coded. Next, we introduce the central features of each model and the procedure for identifying relevant events mentioned in news articles.^c^ We then (1) assess the effectiveness of each coding type for the same data using common metrics—e.g. precision, recall, F1 scores, and AUC/ROC graphs; and (2) compare the results to human coding across cases. Overall, we find that transformer models—both an out-of-the-box GPT-4 model and a fine-tuned BERT model—outperform the semi-supervised dictionary-based classifier bag-of-words approach. Furthermore, GPT-4 performs consistently whereas the reliability of human coding varies substantially with their expertise on a topic. The model’s performance suggests some important refinement to our thinking about prompt engineering. We conclude by identifying topics to consider when incorporating machine coding to the collection of political data.

## The setting

The AMAR data codes information for a representative sample of socially-relevant ethnic groups across the world between 1945–2006 (14). The source material—from news reports to research articles, from books to websites—is all *publicly available*. This makes AMAR’s data collection efforts uniquely transparent and verifiable. The principal drawback with such a strategy, however, is that human coding of this scale is prohibitively expensive and slow—thus making updating and other maintenance untenable. One solution is to draw on advances in computational methods (15)—in conjunction with the use of publicly-available source material. A demonstrably reliable model of the data identification process can reduce the cost of data-coding, while increasing its speed.

Since multiple commercial services collect publicly-available electronic texts, we outsource the first step—creating a corpus of new material relevant to the unit of analysis—to BuzzSumo.^d^ Here, the units of analysis are ethnic groups and for this article, we focus on **African-Americans** in the US and **Dalits** in India in 2020 and 2021. These data are currently **not** coded in the publicly available AMAR data. To retrieve a corpus, we provide BuzzSumo with our ethnic dictionaries and search terms, limiting our results by language (to English) and the defined list of countries.^e^ To ensure that the resulting corpus includes information about the relevant ethnic groups, we also give BuzzSumo a list of newspapers that we know are likely to publish articles about said groups.

To assess accuracy of machine coding of a variable—in this case, **protest**—we rely on commonly-used metrics: (1) *precision*, i.e. does the model identify only the relevant data points?; (2) *recall*, i.e. does the model find the relevant cases?; and (3) *F1*, i.e. what is the overall accuracy score based on precision and recall?^f^

The literature uniformly agrees that the best way to assess accuracy is to compare the predictions from the machine-coded data to the same observations that have also been human-coded (9, 12). The simplified procedure is:

1. Have a research team annotate news articles.
2. Split the data into two sub-samples for training (80%) and testing (20%) the language models.
3. Task the language models to code the same data by learning from the training sample.
4. Assess the accuracy of machine-coded data by comparing human-coded and machine-coded results (on testing sample).

### Human coding

Expert coders are commonly students or research assistants (6, 7) with subject area expertise. We recruited multiple teams (N = 7) of undergraduate research assistants (N=6 students per team on average). Students received class credit for participation. Unlike prior validation assessments, we further trained our experts with coding. In the first meeting, we introduced the project, the AMAR codebook, and the coding process. We walked the students through several example articles (N = 10). We then gave students the above-referenced random sample of news articles. We asked the students to identify whether an article provided sufficient evidence to be coded as about: (1) a specific ethnic group found in the AMAR data, and (2) protest by the subject group. These were independent decisions. We also asked students to substantiate their decisions by providing a quote from the article to support their coding.

For the first week, we assigned every student the same 25-article batch. In the following week, we reconvened for further training where we discussed “controversial” articles—i.e. those that produced split decisions among coders—and adjudicated based on group consensus. If we believed the students still struggled with the coding assignment (only one team struggled), we continued the training by giving every student another 25-article batch. Once the students grasped the assignment, we assigned more articles (N = 50); we also dropped the number of coders per article to four students. Coding happened over multiple semesters. In all, the teams coded 1,718 news articles for this analysis.^g^

Intercoder reliability varied. For example, the ICC among the six coders responsible for labeling news articles related to African Americans ranged from 0.93 to 0.97. For the Dalit articles (four coders), there was less reliability as to whether the article was about Dalits (ICC = 0.48). Note, however, the ICC for protest in the Dalit sample indicated greater reliability (0.84).

We took several steps to remedy low ICC scores. First, we identified potentially problematic categories early on during consensus coding due to the number of emerging “split” decisions. In these cases, second, we returned to the codebook; we went over it with the students to ensure adequate understanding of a given category. Third, we group-coded several articles with the students to verify sufficient understanding. Finally, we allocated more time on split decisions during consensus-coding sessions.

Despite coding articles together as a team and the consensus coding of controversial articles, reliability for the Dalit category remained low. Moreover—compared to the African-Amerian sample—the ICC scores for protest also suffered when the sample was drawn from articles about Dalits. The different ICC scores for the African-American and Dalit categories illustrate that while reliability is high when experts code a familiar category—e.g. African-Americans—it suffers when they code less familiar ones, even when trained. Furthermore, while the largest potential problems may arise when coders categorize “unfamiliar” identity groups, reliability also decreases when categorizing “transferable” concepts (e.g. protest) in unfamiliar contexts.

### Model 1: Newsmap

We use a naive Bayes classifier as our baseline algorithm for comparison to the human coding. The model, *Newsmap*, was originally designed to improve the geolocation of news articles over current gazetteers (16). As a bag-of-words model partially trained on a researcher-provided set of “seed words”, *Newsmap* requires fewer assumptions for generating predictions—thus making it especially useful when researchers are interested in a well-defined set of cases and variables. We derive the seed words to train the model from two sources: (1) directly from the AMAR codebook and (2) from existing news sources used to justify AMAR coding decisions. The *Newsmap* workflow is a trained machine application for coding. Table 1 summarizes the model’s workflow.

**Table 1.** Overview of the *Newsmap* model process.

1. Obtain corpus of relevant documents for analysis.
2. Create dictionary of seed words related to topic of interest.
3. Identify relevant documents containing seed words from dictionary.
4. Classify portion of documents (80% of data) using labeled documents from step 3 as training data.
5. Take model results from step 4 and introduce more unlabeled documents into total data pool.
6. Estimate probability that words in unlabeled data predict each class.
7. Re-estimate classifier from step 4 with results from steps 5 and 6.
8. Repeat steps 5–7 until model converges.
9. Introduce the remaining 20% of human coded data to *Newsmap* for classification.

### Model 2: BERT

We compare *Newsmap’s* performance against models with different architectures, namely *transformer* models. Although bag-of-words models are more transparent and computationally less expensive, they may have less predictive power for large or complex texts—thus making a good comparison for more context-focused transformer models. While bag-of-words models only consider the frequency of terms in a document without regard for context, transformer models weigh the importance of certain words in context. The mechanism for weighing the predictive power of words in context is called *self-attention*, and it allows for both increased speed and precision when completing complex classification tasks.

The first transformer model we evaluate is BERT, which comes pre-trained on a large corpus of documents for a general understanding of language in context (17). Addressing concerns with zero-shot applications, researchers can then fine-tune the model to solve specific tasks, e.g. providing additional labeled texts for classification. We describe the fine-tuning process in the Supplementary material. BERT is effective at several tasks including classification; there is also evidence that it outperforms many other similar models on NLP tasks. Table 2 outlines the modeling process when using BERT. We run BERT in Python via the *transformers* package.

**Table 2.** Overview of the BERT model process.

1. Obtain corpus of relevant documents for analysis.
2. Load BERT model pre-trained on a general corpus of texts.
3. Fine-tune BERT model for specific coding project, using a sample (80%) of human-coded data from the corpus obtained in step 1.
4. Introduce remaining 20% of human coded data to BERT for classification.

### Model 3: GPT

We also consider OpenAI’s GPT models. Like BERT, the GPT family of models is pre-trained on a very large corpus that provides them with a general understanding of language in context. GPT is therefore well-suited for classification tasks, including identifying topics or events in news articles. The literature variously tests on different generations including 3.5 (6, 8) and 4 (7, 9), complicating comparisons. For clarity, we test four generations of GPT models but only discuss the results of GPT-4 here, leaving comparisons to earlier generations for the Supplementary material. The modeling process for GPT and other openAI models is presented in Table 3.

**Table 3.** Overview of the GPT model process.

1. Obtain corpus of relevant documents for analysis.
2. Construct prompts that accurately define coding task for the GPT model to perform.
3. Test accuracy of prompt responses on a small set of data.
3. Pick prompt that performs best and run the OpenAI API request for test sample of articles in corpus.
4. Run final prompt multiple times on test data.

Currently researchers can only fine-tune GPT models up to version 3.5 (18) to perform specific classification tasks. Therefore, we focus on prompt engineering—arguably considered by the literature as the most important procedure when using GPT models—to address reliability and validity concerns with respect to zero-shot applications (3, 8, 19).

To engineer prompts, we simulated text about protest and non-protest events, varying levels of detail and specificity (see Supplementary material for details). The best-performing engineered prompt—both in precision and recall for classifying our data—was:

**Engineered prompt:**  *Classify protest events based on contextual cues. Consider keywords like “protest”, “demonstration”, “rally”, “strike”, “march”, and “sit in”. Protests could be violent or symbolic forms of resistance. Examine contextual information such as location, participants (groups, organizations, activists, advocacy groups, specific communities), event date, and time. Check for motivations, demands, grievances, and the presence of law enforcement when large groups gather for a cause. Use “yes” or “no” to indicate if the article describes a protest event.*

We use the Chat Completions API with this prompt on the same five folds of data used for the other models. Because GPT models can respond differently over multiple iterations to the same prompt, we run the GPT-4 model three times over the five folds (altogether 15 runs) using the same prompt, take the modal output value for each fold, and compare that to the human-coded output. We set the temperature hyperparameter for this set of runs to 0 (no randomness in output). For robustness, we also vary the temperature as recommended in the literature (8) as explained below. We use the *httr2* package (20) in R to interact with the API.

Additionally, as discussed below, we compare the performance of our engineered prompt to the following simple baseline prompt: *“Identify with ‘yes’ or ‘no’ whether the following article (delimited in XML tags) mentions a protest event:*  ⟨article⟩text⟨article⟩”. (For detailed results, see Supplementary material).

Next, we explain the collection and coding process for the data. We use these data to compare the results of Newsmap, BERT, and GPT-4 classification to human coders.

### Data

Our initial set of labeled news articles contained 1,718 unique documents. Of the 1,718 documents, only 454 (26%) observed protest. Such class imbalances are problematic for both training and assessing model performance. This is because the resulting metrics can be biased towards majority-represented classes. To account for this, we down-sample the data when performing fine-tuning and testing so that all 454 observations of protest are present alongside a random sample of 454 “not protest”-labeled texts. The resulting sample contains 908 unique documents evenly split by labels that we use to train and test *Newsmap*, fine-tune and test BERT, and run our prompts on GPT-4.

To compensate for the loss in observations when assessing model accuracy via down-sampling, we run all the models using five-fold cross-validation. *K*-fold cross-validation takes *k* sub-samples of the data and then trains and tests the model on said sub-sample (fold). Each model uses the same indices for each fold to improve result comparability—i.e. observations are the same across models. As is the case when using cross-validation techniques, the final model performance metrics are the average prediction results across the five test sets created for each fold.

## Results

### Five-fold cross-validated results

Table 4 presents the average precision, recall, and F1 scores across the three models. *Newsmap* performed the worst, with lower mean recall and precision scores than the other models. As expected, BERT had greater reliability, with a mean precision of 0.76 (F1 = 0.77). The GPT-4 model with the engineered prompt still outperformed the other models in F1 scores because it has a high recall score of 0.94 (F1 = 0.82).

**Table 4.** Performance metrics across three models for identifying *protest* label in news articles.

Model	Precision	Recall	F1
Newsmap	0.62	0.53	0.57
BERT	**0.76**	0.79	0.77
GPT-4	0.74	**0.94**	**0.82**

Metrics represent average results for models using five-fold cross-validation. Bold numbers indicate model with the highest score for the given metric.

**Fig. 1.**
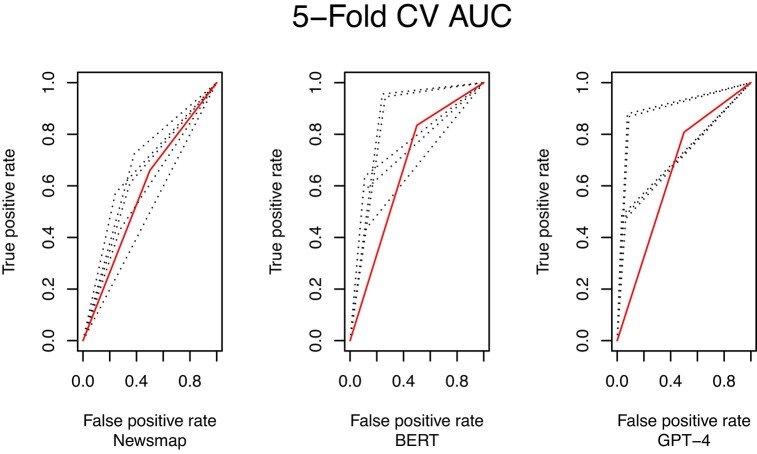
AUC/ROC graphs for three models predicting protest in news articles. Black lines are ROC curve results from individual cross-folds when testing using five-fold cross-validation. Red lines are overall ROC curves. Results generated in *R* (v 4.2.1) using the *cvAUC* package (v 1.1.4).

Figure 1 shows the AUC/ROC curves for each model’s performance across each fold of the five-fold cross-validations. The *y*-axis shows the rate of true positives; and the *x*-axis, the false positive rate. Some folds in the assessment received low accuracy results across all three models (Newsmap, Bert, GPT). This suggests the results are likely a lower-bound for all models because some of the data was harder to classify. The curves suggest similar results to the metrics in Table 4, but they also highlight differences between the approaches. First, BERT has the highest true positive rate of the models—arguably the most important statistic when coding rare events.

### Prompt engineering, temperature, text complexity

We also compare the performance of GPT-4 when using our baseline and our engineered prompt. Interestingly, and contrary to our expectations, Table 5 shows that GPT-4’s ability to classify only true protest events (precision) was consistently better when using the baseline prompt, whereas the model’s ability to detect all protest events in the data (recall) was consistently better with the engineered prompt.

**Table 5.** GPT-4 performance after prompt engineering (average five folds).

	Precision	Recall	F1
Engineered prompt	0.74	0.94	0.82
Base prompt	0.78	0.91	0.83

To test the effect of temperature on model performance (8), we varied the temperature hyperparameter for GPT-4 with the engineered prompt on a sample of articles. The accuracy metrics when the temperature hyperparameter is set at 2 are comparable to those when the temperature hyperparameter is set at 0—thus demonstrating temperature variance did not affect our modeling substantially. However, because this analysis was run on a sample and there is a slight improvement with higher temperature setting, we conclude that this is an important topic for further research.

Finally, to allow for a more direct comparison between humans and the machines in the above test, we fed the machines the exact same articles that the humans were asked to code. To test GPT-4 performance across more or less complex text, which may matter for performance (21), we heavily pre-processed a balanced sample of articles of median length. We did not find evidence that substantial pre-processing improved LLM performance. For further details on all robustness checks, see the Supplementary material.

### GPT-4 performance

Why did GPT-4’s overall accuracy (F1) fail to consistently exceed that of humans—per earlier work (6, 7)? We suggest several explanations. First, our human coders received iterative training—setting a higher, and arguably more realistic, bar for comparison.

Second, because the data are new, we need to rethink the notion of human-coded data as ground truth. We can think of the human coder ICC as measuring the consistency across coders. In contrast, machine accuracy is measured by comparing how well the machines replicate human coding. While ICC scores measure consistency, the variance in scores can indicate the complexity of a coding task, which likely affects the accuracy of human coding. The machines can be internally consistent in their own coding (with GPT-4 Cronbach’s alpha being above 0.90 across iterations). However, when replicating human coding the best they do is capturing around 80% (F1) of cases coded by the humans.

There is a caveat to the presumed quality of human coding captured by the ICC scores. Specifically, humans are only highly consistent when coding well-known categories such as reports about African-Americans. Once we shift to less well-known categories such as classifying reports about Dalits, ICC scores drop as low as 0.48. With improvements in model precision—potentially achieved through multi-shot applications and fine-tuning—transformers may improve in their overall accuracy. Furthermore, the consistency in transformer coding metrics across all cases suggests this class of model may rival human coders when coding cases where humans do not have area expertise ((1), p. 28).

Third, task variance has a substantial effect on LLM performance (9) for reasons not yet well articulated in the literature. Our results showed that the recall metric (false negatives) of LLMs approached that of human coding, while the precision metric (false positives) fared worse. As such, the performance of LLMs varies not only in overall accuracy as noted in the literature, but also with respect to the specific task at hand—e.g. whether the goal is to classify only true positives or to find all positives. Moreover, we found that the baseline (simple) prompt resulted in higher precision in classifying protests, while the engineered prompt consistently had higher recall scores. Addressing calls in the literature for better prompt engineering (3), we add that the need for prompt engineering is likely task dependent. In contrast to the simple classification performed in this article, prompt engineering may be more important for complicated classifications such as conditional statements or multistage classification. Furthermore, the need for prompt engineering likely depends on the desired outcome and is probably more important in early stages of data collection where completeness outweighs concerns with mis-classification.

## Conclusion

These results demonstrate that human validation is still important and necessary to ensure construct validity. However, LLMs may prove useful for helping coders make decisions about new data, especially when coding rare events and/or less familiar contexts. The GPT models, in particular, demonstrated strong performance without fine-tuning. Thus, startup costs are low. Moreover, if coding records already exist, they can be used to calibrate machine coding for improved reliability and validity. In spite of this discussion, there are important caveats to consider.

Notably the internal processing of the best performing models BERT and GPT-4 is opaque; moreover, these models are still prone to error. Even so, the LLMs demonstrate sufficient internal consistency and accuracy with respect to human coding to be an important step in making human coding of data more efficient. Human coders simply cannot process and annotate the same volume of material as the machine. Therefore, researchers may want to consider a reverse workflow where model results are used as a “screener test” to highlight evidence of protest subject to human verification. Furthermore, machine output can be set to reference sources—thus allowing coding decisions to be easily verified. In this way, machine coding can help improve the transparency of output.

Importantly, we remain agnostic about whether we should consider human coding or LLM classification as the “ground truth”. For example, human coders can possess similar biases that lead to similar coding decisions, and thus bolster accuracy metrics. As such, we contend that the two methods are complementary. Together, they should be used for triangulation. When the classifications of the two methods align, we should have greater confidence in their results. Conversely, when classifications diverge, we should proceed with caution and ask why.

Furthermore, variations in human coder reliability indicate that spatial and temporal (22) contextual knowledge is highly relevant for expert-coding accuracy. In contrast, machine coding showed greater consistency across cases. One drawback is, as noted, that the best-performing algorithm—GPT—is also the most opaque. Thus, best practices for a particular task may be subject to experimentation and not necessarily applicable across tasks. All this suggests that the comparison of machine coding to human coding across tasks remains a topic for further study.

Finally, machine-coding methods are easily adaptable. They can address new questions or subject matters not just from different places and times, but also across different units and outcomes. This can potentially facilitate broad spatial and time-series analyses of politics—including at the sub-national level, where definitions and boundaries of groups and concepts change over time (22).

## Supplementary Material

pgae165_Supplementary_Data

## Data Availability

All data and code used for analysis and creating visualizations will be included in publicly available replication files. The replication files will be made available on Harvard Dataverse as “Replication files for Coding with the machines”. Additionally the files will be available on Github at www.github.com/jkbirnir/amar_and_the_machine or www.github.com/jdewar/amar_and_the_machine.

## Notes

a As opposed to multi-shot and fine-tuning, which train models on a subset of the data.

b AMAR is an independent continuation of Ted Gurr’s *Minorities at Risk* data.

c See Supplementary materials for a detailed account of each method.

d Users need to consider selection issues with the curating of sources.

e See Supplementary material for the search terms used to build the corpus.

f See Supplementary material for metric description.

g See Supplementary material for descriptive statistics on the final news articles dataset.
