# Supplementary material for: Coding with the machines: machine-assisted coding of rare event data
Source: PNAS Nexus. 2024 Apr 30;3(5):pgae165. doi: 10.1093/pnasnexus/pgae165 (PMC11102067; doi:10.1093/pnasnexus/pgae165)
Supplement: pgae165_Supplementary_Data [file pgae165_supplementary_data.pdf]

PNAS Nexus, 2024, pp. 1–11

doi: DOI HERE
Advance Access Publication Date: Day Month Year
Paper

PAPER

Supplementary Appendix for Coding with the machines: machine-assisted coding of rare event data

Henry David Overos,[1] Roman Hlatky,[2] Ojashwi Pathak,[1] Harriet Goers,[1] Jordan Gouws-Dewar,[1] Katy Smith,[3] Keith Padraic Chew,[4] Jóhanna K. Birnir[1,*] and Amy Liu[3]

[1]Government and Politics, University of Maryland at College Park, College Park, MD, USA, [2]Political Science, University of North Texas, Denton, TX, USA, [3]Government, University of Texas at Austin, Austin, TX, USA, [4]School of Politics and Global Studies, Arizona State University, Tempe, AZ, USA and [*]To whom correspondence should be addressed: jkbirnir@umd.edu

FOR PUBLISHER ONLY Received on Date Month Year; accepted on Date Month Year

Abstract

Draft

Appendix

This appendix includes descriptions of technical details related to the analysis. While not required for understanding our work overall, this additional information may be helpful for researchers interested in replicating the data collection and modeling strategy. This information also includes an account of the steps taken to ensure the ethical collection of data.

First, we briefly describe the relevant details pertaining to the corpus of newspaper data used for coding.[1]

Next, we account for the calculation of the intra-class correlation coefficient that we use to assess the accuracy of the human coding. This section concludes with an explanation of human subject clearance and by addressing the ethics of our engagement with undergraduate coders.

Before we discuss the Newsmap and Large Language Models (LLMS) outlined in the article, we elaborate on the metrics we used to assess the quality of the machine coding. We subsequently outline a brief formal description of the naive Bayes algorithm, of which *Newsmap* is a variant. After that, we discuss the transformer models (BERT and GPT) used for comparison with the baseline *Newsmap* model in the article. The models include both BERT – a bi-directional transformer created by Google AI – and four variants of the GPT transformer – created by OpenAI. For the GPT models, we discuss the multiple steps we took for rigorous examination of its performance in the classification tasks. First, we compare all four models of GPT (GPT-4, GPT-3.5-Turbo, DaVinci, and Ada); we find that GPT-4 performs the best. Subsequently, we apply GPT-4 on multiple subsets of our data to measure its overall performance. Additionally, we test GPT-4 performance by focusing on three factors—prompt engineering, the pre-processing of datasets, and the temperature hyperparameter. Here, we pay special attention to best practices outlined in the literature.

Description of the data

All-Minorities at Risk

The *All Minorities at Risk* (AMAR) dataset was launched in 2015 to (1) update the original *Minorities at Risk* (MAR) data to address selection concerns[2]; (2) add missing minority and majority groups to a reconfigured sample frame of around 1200 groups; and (3) bring the data in line with current understanding of best practices in data collection design. For additional information about this data, see [2, 1]

[1] BuzzSumo is a cloud-based app used mainly for market research and social media content analysis. Their collection is based on articles/texts that are the most widely shared on social media platforms. The software is used by companies such as Hubspot, by magazines such as *Rolling Stone*, and websites such as Buzzfeed in order to track trending content across the internet. Through BuzzSumo, users input website domains or search terms and receive datasets containing the top stories and most shared posts relating to the search.

[2] Note that the AMAR data frame was created specifically to alleviate concerns about which groups were included in MAR.

© The Author 2024. Published by Oxford University Press. All rights reserved. For permissions, please e-mail: journals.permissions@oup.com

The Data Collected

The final dataset used in the project described here is a corpus containing online news articles written and published in 2020. We began by searching for articles about protest (a topic-based search) and specific racial/ethnic groups by country as represented in the AMAR data.[3] Additionally, we consulted with experts on racial/ethnic politics and developed lists of news outlets that are directed at specific minority group readership.[4] To retrieve a corpus, we provided BuzzSumo with our ethnic dictionaries and search terms. We limited our results by language (article title in English) and the defined list of countries. We then scraped the articles using the URLs provided by BuzzSumo. For the analysis, we excluded any URLs for which the article body could not be retrieved.

Descriptive Statistics and Counts Tables

The following tables present descriptive statistics and counts of human-coded labels for the data set used in the main analysis. The total number of human-coded observations was 1718. Each observation represents one unique article that received human coding. Ethnic/racial group variables were coded as categorical variables and then converted into binary variables, where 1 implies the article was about that group and 0 implies it was not about that group. Additionally, a "none" label was used when no group in the codebook was in the article.

For the variables describing political protest (*Protest*), a 1 represents the variable's presence in the article as described in the original AMAR codebook.

Table 1. Descriptive statistics for human coded documents

Statistic	N	Mean	St. Dev.	Min	Max
Protest	1,718	0.268	0.443	0	1

Table results from full 1718 observation human coded data set.

Table 2. Counts of human coded articles by label.

Label	N
Not Protest	1258
Protest	460

Table results from full 1718 observation human coded data set.

There were 13 distinct racial/ethnic groups and 6 countries present across the human-coded articles. In this project, we focus on two: African-American in the US and Dalits in India. Additionally, there were two *none* categories which were used when human coders did not find evidence or discussion of an racial/ethnic group or a country in the article.

[3] We chose protest as a variable for a topic-based search due to our knowledge of a high amount of protests in 2020. This prior knowledge would allow for an easier qualitative assessment of the model's accuracy.

[4] Moving forward, we intend to consult with country experts when developing corpora related to each racial/ethnic group in each country of study.

Table 3. Crosstable of human coded articles by racial/ethnic group and country.

	IND	other/none	US
afam	0	6	35
dalit	777	19	0
other ethnic	0	242	5
none	5	533	96

Table results from full 1718 observations in human coded data set.

Human coding metric

To assess reliability amongst the human coders, we calculate an intra-class correlation coefficient (ICC) for each of the given coding categories. Specifically, we rely on the average measures consistency ICC (ICC 3,k), which is equivalent to a Cronbach's α reliability score. This form of the ICC assesses the extent to which one coder's score approximates another coder's score, given some degree of systematic error [5]. More practically, consider the *Protest* category: for each human coder, ICC 3,k calculates the mean number of positive articles (i.e., those that mentioned instances of protest) for each coder, and then compares the consistency of these means across coders. Given potential differences in coder characteristics, we chose not to calculate a two-way random effects version of the ICC.

Human subjects and research ethics

The coding by undergraduates was completed at University (name redacted for anonymity) and adheres to the University's Institutional Review Board (IRB) guidelines for review. Specifically, instructions published at URL (redacted for anonymity) clarify that the University's human subject guidelines exempt coding of the data described here from review because as per IRB definition this research does not involve human subjects.[5] Furthermore, the researchers guiding the undergraduate coders strictly adhere to the University's ethical standards in pedagogy relating to compensation (here class credit for instruction in research methods), diversity, inclusion, and overall beneficence in teaching and research.

Machine coding metrics

Because the data we code in this project are new, we can think of the human coder ICC as measuring the accuracy of the human coding (tested across multiple humans) - whereas the machine accuracy is measured by comparing how well the machines replicate human coding.

To measure machine coding performance, researchers often use model accuracy as a measure for prediction problems in machine learning. Using balanced data, accuracy is simply the percentage of correct predictions made by the model.[6] Mathematically, this is denoted as:

[5] Specifically, the IRB uses definitions by the Department of Health and Human Services and the Food and Drug Administration; see section 2.1. These definitions stipulate that to constitute human subject research, the data collected is "about" the individuals involved in the data collection or an individual "who is or becomes a participant in research, either as a recipient of the test article or as a control."

[6] Accuracy measures can be problematic when dealing with imbalanced data, but we ameliorate this problem with downsampling prior to testing.

Draft

$$Accuracy = \frac{(\text{True Positive (TP)} + \text{True Negative (TN)})}{\text{All Predictions}} \quad (1)$$

However, we are interested in not only the percentage of correct scores but also what types of predictions the machine performs better or worse at. Therefore, we evaluate three measures suggested in the literature [15]: Precision, Recall, and F1 Score.

Precision accounts for how well the model does in distinguishing only positive events and avoiding mistakenly labeling cases as positive (false positives). Here, it would measure the performance of our model in correctly identifying the cases that were about African-Americans, Dalits, or protests and how many cases it mistakenly labeled as being about African-Americans, Dalits, or protests. Specifically, *precision* is the ratio of our true positives to all the positives (true positives and the false positives). For instance, a *Precision* score of 0.65 means that when our model predicts a body of text as protest, it is correct about 65 percent of the time. Mathematically:

$$Precision\ (P) = \frac{\text{True Positive (TP)}}{(\text{True Positive (TP)} + \text{False Positive (FP)})} \quad (2)$$

The second measure is *Recall*, which accounts for how well the model does at not missing any positive cases. Thus, recall measures the model's performance in correctly finding *all* the true positives and not labeling any true positives as negatives (false negatives). In our case, it would measure the performance of our model in correctly identifying *all* the cases that were about African-Americans, Dalits, or protests. Specifically, it is the ratio of our true positives to the true positives and the false negatives where the model failed to detect a case. Mathematically:

$$Recall\ (R) = \frac{\text{True Positive (TP)}}{(\text{True Positive (TP)} + \text{False Negative (FN)})} \quad (3)$$

Lastly, we also look at the *F1 Score*, which is a harmonic mean [7] of the Precision and Recall scores.[8] Mathematically,

$$F1 = 2\frac{P * R}{P + R} \quad (4)$$

A high *F1 Score* (closer to 1) is also indicative of high *Precision* and *Recall* values – suggesting a better model performance. While *Precision* measures the extent of error caused by false positives, and while *Recall* looks at the extent of error caused by false negatives, *F1 Scores* give us the relative impact of the two measures for our model performance.

[7] The reason for using harmonic mean, instead of a geometric or arithmetic mean is that harmonic mean tends to penalize extreme values. When the *Precision* and *Recall* values are highly unequal, the harmonic mean will be much lower than either a geometric mean or arithmetic mean.

[8] Using mathematical notation, harmonic mean = 2/[(1/x)+(1/y)]

Description of the Naive Bayes Classifier

Naive Bayes is an algorithm which has been used for classification problems in computer science and machine learning [6, 8]. The model is useful due to its simplicity, treating individual words of a text as features. The following section offers a brief formal description of the model. For further descriptions of the version used for *Newsmap*, see [14].

Formally, given a set of D documents,

$$d_i \in D = \{d_1, \ldots, d_D\}, \quad (5)$$

where d_i is an individual document within the set, most models predict a text's category or categories c_j from a set of possible categories C, or

$$c_j \in C = \{c_1, \ldots, c_C\}. \quad (6)$$

Naive Bayes relies – as its name implies – on Bayes' theorem of conditional probability to predict document classes. The outcome of the model is the most likely class c_j that best describes the document d_i in the set of documents D. This is represented formally as

$$P(c_j|d_i) = \frac{P(d_i|c_j)P(c_j)}{P(d_i)}, \quad (7)$$

where c_j is a specific class for labeling documents and d_i is a specific document in the data.

The model is *naive* because each document is treated like a "bag of words." Imagine State of the Union speeches cut up into individual words and put into bags labeled with the speaker and the year it was delivered. A person viewing these bags would not be able to rearrange the words to reconstruct the speech exactly as it was written. But they could count the frequency of individual words in each bag. *Naive Bayes* assumes that all the words in a text are independent of each other in the document. This means that the probability of creating a text is the product of each words' individual probability or formally:

$$P(d_i|w_1, \ldots, w_n) = P(w_1)P(w_2)\ldots P(w_n). \quad (8)$$

The probability of getting a document – assuming it is of a specific class c_j – is simplified given that the model assumes a document is the product of the probabilities for each individual word (per equation 8). This means that the product of the probabilities for each word ($\prod_{k=1}^{n} P(w_{d_{ik}})$) – where n is the number of words in a document d_i and k is a distinct word w in the document – can be substituted for $P(d_i)$. Formally, this substitution is

$$P(d_i) = \prod_{k=1}^{n} P(w_{d_{ik}}). \quad (9)$$

Given these assumptions about document creation in equation 9, the probability of a document d_i given a class c_j can be rewritten as

$$P(d_i|c_j) = P(d_i) \prod_{k=1}^{d_i} P(w_{d_{ik}}|c_j), \quad (10)$$

because the simplified probability for document generation can be substituted into the equation.

Draft

Adding a "General News" category to Pre-process Data for Newsmap

For the Newsmap analysis, in addition to the protest categories, each dictionary also includes a "General News" class, which uses common terms in most online news articles (words such as *subscribe*, *email*, *sign up*, etc.). These words are not part of the actual articles but exist on the web pages surrounding the texts. For example, the website bbc.com has a section called "related topics" under full articles, that contain other web page article URLs that have related keywords to the current page. The news site Politico has buttons for sharing the news on Facebook, Twitter, and Reddit. These words and links, which are a part of a website's user interface and not the article in question, might be picked up when collecting the data through web-scraping methods. We first removed as many irrelevant words as possible from the data set prior to running the algorithms through normal text cleaning methods. However, to be expected, some forms of these words remained. As a result, they were being scored incorrectly into the protest categories, potentially leading to false positives. To handle these potential false positives, we trained the model to identify common webpage words as the separate "General News" category. Text identified as "General News" can safely be assumed to not be about protest and is treated as such in the analysis.

The variable dictionaries

After data collection, we created dictionaries for each category to be classified using *Newsmap* models. The dictionaries have two sets of categories. The first set identifies the *variables* of interest; and the second set, the *groups* of interest. The variables we used in our test are protest (**prot**) and discrimination (**discrim**).[9] While the AMAR variables can be further disaggregated in the dataset (protest has ordinal subcategories based on the type and scale of group protest while discrimination has nominal subcategories based on *how* groups are discriminated against), in this test case, we take a binary approach to see if a model can identify general protest and discrimination at a similar rate to human coders.

The second set of dictionary terms is related to identifying the *group* of interest in the AMAR data. We created dictionaries of terms related to *African Americans* and *Dalits*. To create these dictionaries, we consulted with case experts and the literature on the topic. All dictionaries are presented in Table 4.

Table 4. Dictionaries of seed words for generating *Newsmap* models

Model	Variable	Dictionary Terms
Binary Protest	Protest	protest*, petition*, vandal*, marched, police*
Ethnic Groups	African American	african_american*, black_american*, black_lives_matter, black*, negro*, colored*
Ethnic Groups	Dalits	dalit*, damai, sarki, kami, dholi, gaine, sonar, lohar, kalwar, chamar, harina, ram, dhobi, bantar, chidimar, halkhor, tatma, musahar, dusadh, pasi, dhanuk, kurmi

Draft

Description of BERT

BERT (Bidirectional Encoders from Transformers) is a pre-trained transformer model designed and implemented by researchers at Google AI [3]. Transformer models are a type of neural network model built to understand (in this case text) data in sequence and context. Simply put, this class of models examine whole chunks of text in order and learn to predict the most likely sequence of words in relation to each other. Additionally, transformers rely on a computational mechanism called self-attention, by which the model is able to recognize patterns in which words are more important in a sentence or text for identifying future words in that text.

BERT is a unique transformer model in that it is *bi-directional*. Most transformer models were originally trained to be uni-directional. This means that the model's attention mechanism only works by looking at the words appearing in the text moving right. BERT allows for self-attention to also examine the words to the left, which has been shown to increase the accuracy of word prediction.

As explained in the main article, BERT is pre-trained on a large corpus of documents first in order to gain a general understanding of language in context [3]. We then down-sampled the data when performing fine-tuning and testing on pre-classified data so that all 454 observations of protest were present alongside a random sample of 454 "not protest" labelled texts. The resulting sample was 908 unique documents evenly split by labels. To account for the loss in observations when assessing model accuracy via down-sampling, we ran BERT using five-fold cross-validation.

In the BERT model training process, several key hyperparameters are chosen to optimize performance. The model is trained over a total of 3 epochs, allowing it to iteratively learn from the dataset in multiple passes. To manage the computational load, the training and evaluation phases are conducted with per-device batch sizes of 16 and 20, respectively. This setup ensures a balance between memory usage and the

[9] In addition to the protest and discrimination categories, each dictionary also includes a "General News" class, which uses common terms in most online news articles (words such as *subscribe*, *email*, *sign up*, etc.). These words are not part of the actual articles but exist on the web pages surrounding the actual texts. For example, the website bbc.com has a section called "related topics" under full articles, that contain other web page article urls that have related keywords to the current page. The news site Politico has buttons for sharing the news on Facebook, Twitter, and Reddit. These words and links, which are a part of a websites user interface and not the article in question, might be picked up when collecting the data through web-scraping methods. We first removed as many as possible words from the data set prior to running the algorithms through normal text cleaning methods, however, some forms of these words remained. As a result, they were being scored incorrectly into the protest and discrimination categories, potentially leading to false positives. To handle these potential false positives, we trained the model to identify common webpage words as the separate "General News" category. Text identified as "General News" can safely be assumed to not be about protest or discrimination and is treated as such in the analysis.

speed of computation. The initial learning rate is set at 5e-5. A warmup phase of 100 steps is included to gradually ramp up the learning rate. To mitigate overfitting, a weight decay of 0.01 is applied, adding a regularization term to the loss function. Logging is configured to occur every 100 steps, offering insights into the model's performance and facilitating adjustments as needed. The evaluation strategy is set to 'steps', allowing for frequent evaluations within the training process to closely monitor the model's progress and effectiveness on unseen data.

Description of GPT

The second alternative set of models we evaluate are four of OpenAI's GPT models. Like BERT, the GPT family of models is pre-trained on a very large corpus of documents that provides them with a general understanding of language in context. GPT is therefore well suited for classification tasks, including identifying topics or events in news articles. We test four generations of GPT models: GPT-4 (gpt-4-0613), GPT-3.5-Turbo (gpt-3.5-turbo-0613); Davinci (text-davinci-003); and Ada (text-ada-001). These models differ in how they handle complex tasks and produce accurate outputs. The most important difference between the models affecting their performance in classification tasks is their ability to understand complex language structures. The GPT-4 (specifically gpt-4-0613) and GPT-3.5 models - GPT-3.5-Turbo and Davinci, specifically gpt-3.5-turbo-0301 and text-davinci-300 - are instruction-tuned models that were trained by Open AI using Reinforcement Learning with Human Feedback [10]. The models were built iteratively by Open AI, with human coders assessing which of multiple responses to the same prompts was best. Each model was then fine-tuned by Open AI researchers on a compilation of these best responses [10].

GPT-4 builds on GPT-3.5-Turbo, which builds on the Davinci model. Both the GPT-4 and GPT-3.5 are optimized for chat responses by Open AI. The Ada model is part of the legacy GPT-3 series of models. Although it is not as capable as the GPT-4 and GPT-3.5 models at understanding complex language prompts, it is far more efficient and less costly than these later models.

The article reports on our tests of the performance of GPT-4 model for classification tasks. In this supplementary appendix, to show why we report only on the best performing GPT-4 in the article, we also report on the performance of earlier versions under the sub-section titled "Choosing the GPT Model: Comparing GPT-4, GPT-3.5-Turbo, Davinci, and Ada, Baseline Prompt".

Modeling decisions

There are several important parameters that researchers can control when working with OpenAI's GPT models. In an attempt to maintain consistency in output, we changed two parameters - *max_tokens* and *temperature* - from their defaults.

The *max_tokens* parameter controls the maximum number of tokens the model will output. Tokens are representations of text that are meaningful to the GPT models. These are common sets of characters found within the corpus on which the models are trained that are translated into a format the model can efficiently understand.[10] Our desired output from the model was either "Yes" (if a protest event was detected) or "No" (if one was not). Both "Yes" and "No" are represented by one token. For all of the GPT models, we restricted the maximum number of tokens to one. This discourages the model from outputting a more verbose response and facilitates systematic interpretation of the models' predictions and evaluations of their performance.

GPT-4 also limits the number of tokens (or length of text to be read) per request to the chat completions API. Specifically, a user can initially only send 8192 tokens of texts per request to the chat completions API.[11] Each token contains about 3-4 characters of words. For example, the word "The" would be considered one token by GPT-4. Due to this, we had to limit the number of characters the API could read per article. Therefore, in the main analysis, we limited the number of characters to be read by the API to 4000 characters. This allowed us to seamlessly process our requests through the API. We are not worried about the effect of this on GPT-4's accuracy in predictions because most news articles fall within this word limit. Indeed, the median number of tokens per articles in our data was just over 800 (3200 characters). A potentially more concerning problem is the inclusion of irrelevant material in an article that might increase its length artificially. We address this issue through pre-processing as noted in the article.

The *temperature* parameter describes the sampling temperature used by the model. This determines the degree of randomness adopted by the model for its output. It is useful to think of this parameter as describing the degree of creativity the model is allowed to exhibit. A higher temperature will produce a more random output. This parameter takes values from zero to two. We rely on the existing literature on GPT models to make sure that we are following best practices with respect to testing the effect of temperature on output. Most recent publications either run their models at both a low temperature setting and a high temperature setting and compare the results, or they suggest to run the models on both temperature parameters to check validity and consistency of the results [11, 4, 12, 13]. For this article, we set it to zero (completely nonrandom) to discourage the model from outputting any response other than "Yes" or "No". We also ran the models three times to check the consistency of GPT-4 annotations. Finally, we examine whether our results are sensitive to temperature on a sample of 100 articles by running the GPT-4 model on two different temperature parameters—0 and 2. We also ran the models three times for each temperature parameter to check the reliability of GPT-4 annotations. We conclude that the temperature setting did not make a difference on the annotations from GPT-4 models. The findings for this robustness check are discussed under the section titled "What about the Temperature Parameter?: Robustness Check on 100 articles" in this Supplementary appendix.

Choosing the GPT Model: Comparing GPT-4, GPT-3.5-Turbo, Davinci, and Ada, Baseline Prompt.

In this section, we examine the performance of baseline prompts across all GPT models. We find that the model that performs the best is GPT-4. Due to this, in the main article and subsequent sections in this appendix we only compare the overall performance of our models between the baseline prompt and engineered prompt using GPT-4. The findings and

[10] One token tends to represent around 4 characters in standard written English.

[11] As users pay for analysis their maximum token allotment increases.

Draft

robustness checks we performed, comparing the two prompts, using different datasets are discussed in the subsequent sections.

Table 5. Performance metrics across four GPT models for identifying *protest* label in news articles.

Model	Precision	Recall	F1
GPT-4	0.712	0.741	0.726
GPT-3.5-Turbo	0.715	0.668	0.690
Davinci	0.962	0.192	0.320
Ada	0.265	0.374	0.310

The GPT-4 model produced the best overall results of the four GPT models tested. The recall score of 0.741 and precision score of 0.712 suggests that the model performed well at detecting protest events in the text. The GPT-3.5-Turbo model was more precise (with a precision score of 0.715), but had worse recall (with a score of 0.719). On balance - represented by the F1 score - the GPT-4 model outperforms the GPT-3.5-Turbo model. The Davinci model tended to predict "no protest" for a given news article. This is evident from its precision score of 0.962 and very low recall score of 0.192. Finally, the Ada model performed worse than chance. Further, it produced inconsistent outputs. Despite being explicitly prompted to return either "Yes" or "No", 17.7 percent of returns were other strings from which we were unable to determine the model's prediction.

Once we determined which model performed the best using a simple prompt (*Prompt 1*, detailed below), we tested how that model (GPT-4) performed with two different simple prompts.

Prompt 1:*Identify with 'yes' or 'no' whether the following article (delimited in XML tags) mentions a protest event: ¡article¿text¡/article¿*

Prompt 2:*Identify with 'yes' or 'no' whether the following article (delimited in XML tags) mentions a protest, riot, demonstration, or march: ¡article¿text¡/article¿*

Table 6. Performance metrics across two prompts fed to GPT-4 model for identifying *protest* label in news articles.

Model	Precision	Recall	F1
Prompt 1	0.712	0.741	0.726
Prompt 2	0.711	0.719	0.715

Both prompts were equivalently precise. When they predict that an article mentions a protest event, both are correct 71 percent of the time. However, the second prompt has lower recall than the original prompt: it correctly identifies only 72 percent of all relevant articles (compared to 74 percent). On balance, the original prompt is better able to identify articles that mention protest events. This is the baseline prompt we referenced in the paper.

GPT Prompt engineering

In the paper, we compare the performance of LLMs to that of human coders to determine the viability of using LLMs for data annotation. In order to test the performance of the GPT-4 model, we spent a lot of time and effort in engineering the prompt that would yield the most accurate response because prompt patterns are a primary component to engineer effective prompts [16]. When using GPT models, researchers must construct a prompt written in plain language – asking the GPT model to generate text based on the prompt input. We test several different prompts, varying in levels of detail and specificity. Every prompt followed best practices as outlined in the literature [17, 9] in that it was clear, specific, and simple. Our main challenge revolved around ensuring that the model precisely understood that we were searching for evidence of group protest. We drew on lessons learned from our work with dictionaries to test different combinations of words and phrases that would identify protest events and not falsely identify other events or phrases.

Further, we only used the model to perform a simple task: identify whether or not the news article referenced a protest event. Although GPT models (particularly the more advanced models) can also perform more complex tasks, we produced far better results more efficiently and in a more cost-efficient way by splitting complex tasks such as identifying groups and protest events into more simple subtasks. Additionally, we decided to test prompts that mimicked the instructions provided to human coders, suggesting that the model "pretend" to be a research assistant making coding decisions. This also allows us to compare directly with the performance of the human coders who independently identified groups and protest events in the data.

Finally, we included very clear instructions in our prompts that identified the boundaries of our news article. This helped ensure that the model identified all the relevant text to classify. Further, we provided explicit instructions regarding the output we desired: a "yes" or a "no". This, in conjunction with our setting of the maximum tokens parameter to one, ensured that the model produced output that could be systematically examined. More verbose outputs would have been difficult to parse.

For a baseline comparison we also ran the model with a simple prompt without significant engineering. We call this the baseline prompt for clarity.

The baseline prompt:

- Baseline prompt: *Identify with 'yes' or 'no' whether the following article (delimited in XML tags) mentions a protest event: ¡article¿text¡/article¿*

For prompt engineering of our 3 alternative prompts, we rely on Open AI's guidelines for prompt engineering [9] in addition to the guidelines outlined in the literature [17]. Based on the literature, we focus on six factors relevant to prompt engineering: (1) writing clear instructions and adding details (like providing examples or steps that need to be followed to complete the task); (2) providing reference text because LLMs can easily fake answers; (3) allowing the model to take its time and provide reasoning for answers; and (4) measuring performance and modifying the prompt accordingly.

To ensure that the model correctly uses article context to distinguish "political" protest from the use of the term protest in non-political contexts, we simulated 7 news articles with varying levels and types of context to test the prompts. We tested the three engineered prompts to identify protest events using GPT-3.5-Turbo (the version that does not require an API). Subsequently, we chose the prompt that performed the best.

The three prompts that we examined were:

Draft

- Prompt 1: Act as a helpful assistant who annotates texts for a living. A newspaper article refers to a protest event when it mentions words like “protest” or “rally” or “blockade” or “sit-in” or “outcry” or “verbal opposition” or “symbolic resistance” or “peaceful protest”. Alternatively, the newspaper articles could also use plural forms of the keywords or synonyms of the keywords provided here. A protest event could be initiated against an issue or in support of an issue that affects a group of people. As an annotator, your job is to identify with ‘yes’ or ‘no’ whether the article mentions a protest event.
- Prompt 2: From now on, act as a helpful assistant that annotates texts for a living. We are a group of researchers interested in identifying protest events around the world. We rely on newspaper articles to identify such events. In general, a protest could be peaceful (example: rally, sit-in, blockade, hunger protest, verbal condemnation) or it could be violent (riots). Since it is an event, the newspaper article usually describes a gathering of people or a statement made to the public against a social issue or a policy issue. People could protest against a government, a government official, corporations, and policies. For example, the Occupy Wall Street protests were against corporate practices and the lack of regulations that led to the housing bubble in 2007-2008. We are interested in public protests that shed light on social issues and/or policy issues. Protest could also include collective action to achieve specific demands. Therefore, when you annotate, rely on the contextual cues. Finally, identify with ‘yes’ or ‘no’ whether the following article mentions a protest event.
- Prompt 3: Classify protest events based on contextual cues. Consider keywords like protest, demonstration, rally, strike, march, and sit in. Protests could be violent or symbolic forms of resistance. Examine contextual information such as location, participants (groups, organizations, activists, advocacy groups, specific communities), event date, and time. Check for motivations, demands, grievances, and the presence of law enforcement when large groups gather for a cause. Use ‘yes’ or ‘no’ to indicate if the article describes a protest event.

We tested the prompts in the GPT-3.5-Turbo interface because we wanted a quicker response from ChatGPT to measure the efficacy of our prompts for two reasons. First, we could enter simple sentences that included terms related to protests with varying levels of contextual cues and keep repeating the process to check the consistency of its responses. Second, GPT-3.5-Turbo is available for free, which meant we could ask GPT for its reasoning for each response. During this process we could then modify the wording of our prompt for additional clarity, simplicity, and specificity. Additionally, we wanted to be mindful of the fact that human coders rely on context in addition to coding guidelines. Thus, we wanted to test if the prompts worked better in shorter texts if we provide enough contextual cues. For instance, with two of our prompts, GPT-3.5-Turbo categorized our second article on the list below as not protest, citing that it was a personal protest. Also, it correctly categorized the third article on the list as protest because it mentioned a rally and mentioned an actress participating in the rally to condemn governmental inaction. Using these simulated articles, along with chat-GPT’s explanation of its coding decisions, we learned how to change the wording of our prompts and modify the cues provided to make the prompt more effective in eliciting a correct response from the model. The simulated article text that we used was as follows:

- 53 MPs sat in front of the parliament today to indicate their disagreement against the new bill that makes abortion illegal. It is a symbolic demonstration to condemn the government.
- Seema protested when her dad told her she cannot eat sweets anymore.
- Swara Bhasker, an Indian actress participated in the Delhi peaceful rally today condemning the Delhi government for its inability to take action against religious violence.
- There is a rally in Washington Square.
- A peaceful rally was held in Madison Square Garden against the use of firearms in movie sets.
- SAG union has been on strike to negotiate better pay for crew members.
- The audience protested and booed when the referee gave a yellow card to Messi
- Activist Govinda Gyawali protested in front of the Teaching Hospital against the increasing medical expenses that are incurred by citizens. More people have been joining him since he started his hunger strike 3 days ago.

Of the 3 prompts, prompts 2 and 3 worked the best for inducing the model to correctly identify the protest events and non-protest events from the simulated articles. GPT-4 also charges per the number of tokens used in the prompt. One of our objectives is to make the GPT-4 replication accessible at the lowest cost possible. Since the less verbose third prompt worked just as accurately as the second prompt in classifying protest events, we decided to move forward with prompt 3 for the annotation task we perform in this article.

GPT-4 model performance across prompts, downsampled balanced five-fold data

We use the same downsampled data (908 observations) that we used to test our BERT model to assess model performance of the GPT models. As aforementioned, we downsample to create a balanced dataset that has equal number of protest and non-protest events (coded by human coders). To account for the loss in observations when assessing model accuracy via down-sampling, we ran GPT-4 on 5-fold cross-validation test datasets that were also used for the BERT model. We use the same 5-fold test sets to make the GPT-4 results comparable to the other ML methods we use in this paper.

For running the GPT-4 model, we use the 5 different folds of test sets. Three of these sets have 182 observations and two of them have 181 observations. The models were run 3 times on each of the five test sets that we created. The temperature parameter was set at 0 for a more deterministic model.[12]

Baseline prompt

To isolate the effect of prompt engineering on the performance of the model, we first ran chatGPT-4 using the baseline prompt over the data. Table 7 shows the performance of GPT-4 baseline

[12] We, however, test the effect of temperature on the results from GPT-4 model on a sample of 100 observations in our dataset. The findings for the experiment are discussed in the subsequent sections of this Appendix.

Draft

prompt across five different folds of test sets and the average model performance:

Table 7. GPT-4 Performance for Baseline Prompt

Metric	Fold1	Fold2	Fold3	Fold4	Fold5	Avg
Sensitivity	0.89	0.86	0.93	0.90	0.96	0.91
Specificity	0.92	0.92	0.55	0.54	0.59	0.71
Pos Pred Value	0.92	0.92	0.67	0.67	0.70	0.76
Neg Pred Value	0.89	0.87	0.89	0.84	0.93	0.89
Precision	0.92	0.92	0.67	0.67	0.70	0.78
Recall	0.89	0.86	0.93	0.90	0.96	0.91
F1	0.91	0.87	0.78	0.77	0.81	0.83
Prevalence	0.5	0.5	0.5	0.5	0.50	0.5
Detection Rate	0.45	0.43	0.47	0.45	0.48	0.45
Detection Prev.	0.48	0.47	0.69	0.68	0.68	0.60
Balanced Acc.	0.91	0.89	0.74	0.72	0.77	0.81

Engineered prompt

In order to assess whether prompt engineering makes a difference in the overall model performance in classification of protest events, we also ran GPT-4 using the engineered prompt over the balanced data.

The following Table 8 provides the results for GPT-4 models using the engineered prompt (3) across five different folds of test sets and the average model performance:

Table 8. GPT-4 Model Performance After Prompt Engineering

Metric	Fold 1	Fold 2	Fold 3	Fold 4	Fold 5	AVG
Sensitivity	0.91	0.92	0.96	0.93	0.97	0.94
Specificity	0.87	0.88	0.46	0.49	0.48	0.64
Pos Pred Value	0.87	0.88	0.64	0.65	0.65	0.74
Neg Pred Value	0.91	0.92	0.91	0.88	0.94	0.91
Precision	0.87	0.88	0.64	0.65	0.65	0.74
Recall	0.91	0.92	0.96	0.93	0.97	0.94
F1	0.89	0.90	0.77	0.77	0.78	0.82
Prevalence	0.5	0.5	0.5	0.5	0.5	0.5
Detection Rate	0.46	0.46	0.48	0.47	0.48	0.47
Detection Prev.	0.52	0.52	0.75	0.72	0.74	0.65
Balanced Acc.	0.89	0.90	0.71	0.71	0.73	0.79

Comparing the results in Table 7) and Table 8) illustrates that adding more contextual cues to the prompt did not make much of a difference in the overall performance of the model. Interestingly, the precision score with the baseline prompt (the proportion of positive identifications that were actually correct) is higher than the precision score with the engineered prompt. The recall score (the proportion of all relevant instances that are correctly identified) is lower for the baseline prompt than for the engineered prompt.

GPT-4 and pre-processed data across prompts

For purposes of comparison between humans and the machines, we originally fed the machines the exact same articles that humans were asked to code without pre-processing the underlying data. This meant that some of the articles contained irrelevant material such as advertisements, a video, and a handful of articles that had English language headlines had article bodies in a non-English language. Whereas the human coders could troubleshoot by ignoring irrelevant materials, watch the video, and use translators to gauge the content of the text, such extraneous material simply confuses the machine algorithm.

To test the effect of pre-processing on GPT-4 performance we select a subset of 378 of articles with 189 positive protest events and 189 negative protest events using chat GPT-4. The subset was created from the 908 downsampled/balanced articles that we used in the analyses. We filter the articles to only include articles with less than 832 tokens (median number of tokens in the dataset)[13]. Once we manually cleaned the articles to remove advertisements and irrelevant components (for example, Share icon or More related news icons), we checked the number of articles about protest events and non-protest events. After extensive manual pre-processing, we were left with 189 non-protest event articles (as coded by humans) and over 190 protest event articles (as coded by humans). To make the final dataset balanced, we sample 189 positive protest event articles and used the full set of 189 non-protest event articles, which resulted in a dataset with 378 pre-processed articles. The results are shown in Figure 2, GPT-4 AUC on 387 cleaned articles, in the main article. In this iteration of the GPT-4 prediction, we manually clean the article body to clear advertisements, and irrelevant phrases (including website options, sign up options, copyright messages, cookie messages, etc.).

The results below in Table 9 are the full accuracy output when running GPT-4 classification with 0 temperature on 378 articles that were highly pre-processed. We perform two different analyses with our GPT-4 models. First, we ran the model with our baseline prompt, then we compared the model predictions to the human coding to calculate the overall accuracy of the model using the baseline prompt on manually pre-processed articles. We followed a similar process for the same dataset with engineered prompt as well. Table 9 presents the full output of accuracy metrics for both the baseline and the engineered prompts.

Table 9. Comparing the Baseline Prompt and Engineered Prompt on Manually Preprocessed Data

Metric	Baseline	Engineered
Sensitivity	0.89	0.90
Specificity	0.71	0.68
Pos Pred Value	0.76	0.74
Neg Pred Value	0.87	0.88
Precision	0.76	0.74
Recall	0.89	0.90
F1	0.82	0.81
Prevalence	0.5	0.5
Detection Rate	0.45	0.45
Detection Prevalence	0.59	0.61
Balanced Accuracy	0.80	0.79

Most notably the relative performance of the baseline and the engineered prompt remain consistent across more and less

[13] The mean number of tokens for the articles was 1034. However, we only had about 50 articles with number of tokens exceeding 832 tokens, which is why we went with the median number of tokens for the analysis.

Draft

pre-processed datasets. To show this more clearly, Table 9 depicts different accuracy results by data used and GPT-4 prompt. The baseline prompt is consistently more precise (yields less false positives), and the engineered prompt has a better recall (yields less false negatives) across more and less cleaned data, and shorter articles. Figure 1 visualizes the GPT-4 results on 378 pre-processed articles. The graph on the left shows the AUC curve with the engineered prompt. The graph on the right shows the AUC curve with the baseline prompt.

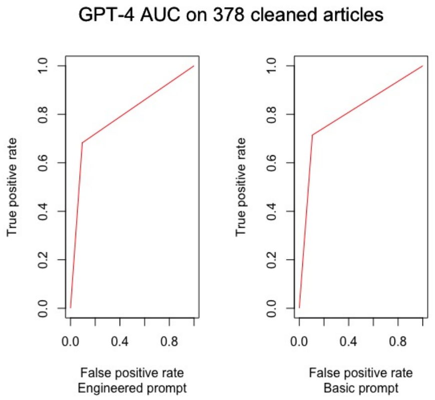

Fig. 1. AUC graphs of GPT-4 results on 378 pre-processed articles. The left graph shows results with the engineered prompt and the right graph shows results with the baseline prompt.

Finally, Table 10 provides accuracy results across different sub-samples of our data. The table provides overall performance of the GPT-4 models using both baseline and engineered prompts on multiple sub-samples of the articles in our data. As can be seen in the table, the baseline prompt is more precise in classifying articles and the engineered prompt has a better recall across all models used in this analysis.

Table 10. Accuracy results across different sub-samples of article data. Results are all from GPT-4 models with varying comparison data and prompt styles. Bold numbers indicate the highest result for that metric.

Data	Prompt type	Precision	Recall	F1
Uncleaned (n=902)	Engineered	0.74	0.94	0.82
Uncleaned (n=902)	Baseline	0.78	0.91	0.83
Median length articles (n=100)	Engineered	0.8	**0.93**	0.86
Median length articles (n=100)	Baseline	**0.87**	0.92	**0.89**
Highly preprocessed (n=378)	Engineered	0.74	0.9	0.81
Highly preprocessed (n=378)	Baseline	0.76	0.89	0.82

What about the Temperature Parameter?: Robustness Check on 100 median length articles

To subject the model to a temperature robustness check as suggested in the literature [12], we selected a subset of 100 articles of median length from our main dataset. For this purpose we filtered the articles to only include articles with more than 200 characters or 50 tokens to make sure the body was sufficiently long to constitute an actual article.[14] The resulting dataset had 425 non-protest events (as coded by humans) and 384 protest events (as coded by humans). Wefiltered our data further to only include articles that had more than 50 tokens but less than or equal to 832 tokens.[15] One reason for this decision was to make sure that the entire articles were read by the machine without any errors (see earlier discussion of chat-GPT token limitations). Once we filtered the data to only include articles with 832 tokens or less, the samples (of 50 each) were randomly drawn from 217 articles that were categorized as not protests and had less than 832 tokens (out of 425 total non-protest event articles) and from the 188 articles that were identified as discussing protest events by our human annotators and had less than 832 tokens (out of 384 total protest event articles). The final pre-processed sample contained 100 observations, 50 with negative protest events and 50 with positive protest events.

To ensure that the shorter articles contained relevant text (as opposed to irrelevant material such as advertisements), we pre-processed the data minimally as follows: First, using customized stop-phrases, we eliminated superfluous text from the data, such as advertisements or copyright statements. Then, we filtered the data to only include article bodies in English.

We used the third engineered prompt (same as in the article) to test GPT-4's predictions at different temperature settings. First, we ran the GPT-4 model with the third prompt 3 times at 0 temperature. We used Cronbach's alpha to measure intercoder reliability of the GPT-4 model annotations at a temperature setting of 0. The Cronbach's alpha for this iteration was above 0.9, which means that the predictions were consistent each time we ran the model at 0 temperature. Second, we ran the GPT-4 model with the third prompt 3 times at a temperature setting of 2. The Cronbach's alpha for this iteration was also above 0.9, which means that the GPT predictions were also consistent within this iteration even though we set a higher temperature to allow for for a more diverse prediction. We use the mode of the three GPT-4 model predictions at each temperature to calculate the accuracy of GPT models in comparison to human coders. Finally, we created two confusion matrices, the first one containing the predictions (precision, recall and F1) from the GPT-4 model with the lowest temperature (0) and the second one for the GPT-4 model with the highest temperature (2). The table below summarizes the performance metrics.

Table 11. Performance of GPT models using 100 pre-processed articles, varying temperature

Model	Accuracy	Precision	Recall	F1
GPT-4 at 0 temp	0.87	0.8	0.93	0.86
GPT-4 at 2 temp	0.88	0.83	0.96	0.88

For the deterministic model where the temperature parameter was set at 0, the GPT-4 model correctly identified 47 non-protest events and 40 protest events. Of the 10 articles that were identified as protest by human coders, GPT identified

[14] Each token has about 4 characters of words.

[15] The median number of tokens per article in our dataset was 832, which is the reason why we limited the tokens to 832.

Draft

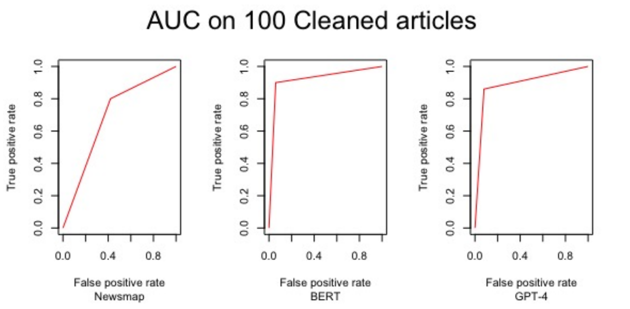

Fig. 2. AUC/ROC graphs for models tested on 100 cleaned, balanced articles.

them as non-protest events. This is parrticularly interesting because upon further investigation, we found evidence that GPT models might be predicting based on the context provided in the articles. For example, one of the articles that human coders identify as describing a protest event measures support for the Black Lives Matter movement by race in the US. The GPT model correctly predicts this as a non-protest event. Likewise, another article that was identified as a protest event by human coders but a non-protest event by GPT compares Trump's campaign rallies to Klan rallies but it does not mention a protest event. Therefore, the GPT model, in some cases, performs better than human coders based on the prompt we provided.

For the random model where the temperature parameter was set at 2, the GPT-4 model correctly identified 48 non-protest events and 40 protest events. The GPT prediction pattern here is very similar to the deterministic model. There is only one case where the random model disagrees with the deterministic model and agrees with human coders, where the human coders correctly identify a non-protest event. This could be further indication that using both deterministic and non-deterministic models for classification tasks can ensure more accurate annotations at a lower cost.

Noticing that the performance of the model improved when the articles were thoroughly manually cleaned, we decided to compare all models on this small sample of 100 cleaned articles. The results highlight the importance of data quality when using any statistical learning method, bag-of-words or otherwise. As can be seen in the AUC/ROC graphs in Figure 2, all models produced significantly higher results when the test set is comprised only of cleaned texts. BERT and GPT-4 dramatically improve in performance metrics. with F1 scores, precision, and recall all above 0.8.

For GPT-4, the results did not change much when we used different temperatures or prompts. Consequently, we did not pursue this line of inquiry further.

Conclusion

The objective of this article and supplementary appendix is to contribute to the understanding of validation of machine classification. In addition to the concerns detailed in the paper the literature highlights a number of issues where more research is needed. Among these is a concern with various types of bias ranging from predominant use of English language sources to biased stereotypes in texts [**?** 17, 7**?**]. While we do not examine bias in this paper we point to this as an important direction for future studies.

Draft

References

1. Jóhanna K. Birnir, David D. Laitin, Jonathan Wilkenfeld, David M. Waguespack, Agatha S. Hultquist, and Ted R. Gurr. Introducing the AMAR (All Minorities at Risk) Data. *Journal of Conflict Resolution*, 62(1):203–226, 2018.
2. Jóhanna K Birnir, Jonathan Wilkenfeld, James D Fearon, David D Laitin, Ted Robert Gurr, Dawn Brancati, Stephen M Saideman, Amy Pate, and Agatha S Hultquist. Socially relevant ethnic groups, ethnic structure, and amar. *Journal of Peace Research*, 52(1):110–115, 2015.
3. Jacob Devlin, Ming-Wei Chang, Kenton Lee, and Kristina Toutanova. BERT: Pre-training of Deep Bidirectional Transformers for Language Understanding. arXiv:1810.04805v2 [cs.CL], 2019.
4. Alizadeh M. Gilardi, F. and M. Kubli. Chatgpt outperforms crowd-workers for text-annotation tasks. arXiv:2303.15056v2 [cs.CL], 2023.
5. Terry K Koo and Mae Y Li. A guideline of selecting and reporting intraclass correlation coefficients for reliability research. *Journal of chiropractic medicine*, 15(2):155–163, 2016.
6. David Lewis and Marc Ringuette. A Comparison of Two Learning Algorithms for Text Categorization. 1996.
7. Clotilde Napp. Gender stereotypes embedded in natural language are stronger in more economically developed and individualistic countries. *PNAS Nexus*, 2, 2023.
8. Kamal Nigam, Andrew McCallum, Sebastian Thrun, and Tom Mitchell. Learning to Classify Text from Labeled and Unlabeled Documents. 39:8, 1998.
9. OpenAI. Open AI developer platform: Prompt engineering. *https://platform.openai.com/docs/guides/prompt-engineering*, 2023.
10. Jeff; Jiang Xu; Almeida Diogo; Wainwright Carroll; Mishkin Pamela; Zhang Chong; Agarwal Sandhini; Slama Katarina; Ray Alex; Schulman John; Hilton Jacob; Kelton Fraser; Miller Luke; Simens Maddie; Askell Amanda; Welinder Peter; Christiano Paul; Leike Jan; Ouyang, Long; Wu and Ryan Lowe. Training language models to follow instructions with human feedback. *OpenAI*, 2022.
11. Samuel Wolken Neil Fasching Pangakis, Nicholas. Chatgpt-4 outperforms experts and crowd workers in annotating political twitter messages with zero-shot learning. arXiv:2306.00176v1 [cs.CL], 2023.
12. M.V. Reiss. Testing the reliability of chatgpt for text annotation and classification: A cautionary remark. arXiv:2304.11085v1 [cs.CL], 2023.
13. Usman Naseem Mehwish Nasim Thapa, Surendrabikram. From humans to machines: Can chatgpt-like llms effectively replace human annotators in nlp tasks? *Association for the Advancement of Artificial Intelligence*, Workshop Proceedings of the 17th International AAAI Conference on Web and Social Media, 2023.
14. Kohei Watanabe. Newsmap: A semi-supervised approach to geographical news classification. 6(3):294–309.
15. Kohei Watanabe and Yuan Zhou. Theory-Driven Analysis of Large Corpora: Semisupervised Topic Classification of the UN Speeches:. *Social Science Computer Review*, February 2020.
16. Jules White, Quchen Fu, Sam Hays, Michael Sandborn, Carlos Olea, Henry Gilbert, Ashraf Elnashar, Jesse Spencer-Smith, and Douglas C. Schmidt. A prompt pattern catalog to enhance prompt engineering with chatgpt, 2023.
17. Zhou K. Li J. Tang T. Wang X. Hou Y. Min Y. Zhang B. Zhang J. Dong Z. Zhao, W.X. and Y. Du. A survey of large language models. arXiv:2303.18223v13 [cs.CL], 2023.

adjustbox

Draft
